# An Insight into Biology, Function and Pest Management Guidance of Gut Microbiota in *Spodoptera frugiperda*

**DOI:** 10.3390/insects16121237

**Published:** 2025-12-08

**Authors:** Xiao-Rui Yan, Jia-Ni Li, Ze-Yang Sun, Chun-Cai Yan

**Affiliations:** Tianjin Key Laboratory of Conservation and Utilization of Animal Diversity, College of Life Sciences, Tianjin Normal University, Tianjin 300387, China; yanxr@tjnu.edu.cn (X.-R.Y.); ljn18602604560@163.com (J.-N.L.); skysunzeyang@tjnu.edu.cn (Z.-Y.S.)

**Keywords:** *Spodoptera frugiperda*, gut microbiota, symbiosis, insect pest management

## Abstract

Gut microbial community research has garnered considerable attention due to its pivotal role in shaping diverse insect hosts’ life-history traits, with key studies confirming that insect gut microbiota is vital for core physiological processes including growth, nutrient metabolism, nitrogen fixation, pheromone biosynthesis, and environmental adaptation. Findings highlight that integrating insect ecology understanding with gut microbial community characterization is indispensable for innovative pest management strategies. Notably, *S. frugiperda*, a globally destructive agricultural pest causing substantial annual economic losses, has been extensively investigated. Its host range is extremely wide, from staple food crops such as corn and rice to various cash crops. This review systematically synthesizes the prevalent diversity of its gut’s dominant microbes, clarifies the important mechanism of gut microbiota in host stress adaptation (providing direct evidence for explaining the pest’s stress resistance formation), draws the key conclusion that host–microbe interaction mechanisms can serve as key pest management targets to guide more targeted control technologies. We also discuss current limitations such as inadequate microbial function verification and unclear host–microbe molecular mechanisms while outlining future directions including focusing on microbial community dynamic changes under multiple environmental stresses and functional microbe screening and application.

## Simple Summary

*Spodoptera frugiperda* is a globally distributed and highly destructive agricultural pest that causes substantial annual economic losses. Its gut harbors a diverse microbial community shaped by both external and internal factors, and these symbiotic microbes play important roles in the pest’s physiological processes and stress adaptation. This review focuses on the prevalent microbial diversity in the *S. frugiperda* gut, the function of these microbes in the host’s stress adaptation, and the potential value of this knowledge for pest management. It also addresses current research limitations and outlines future directions, aiming to clarify the interaction mechanisms between the gut microbiome and *S. frugiperda*, thereby providing guidance for developing more effective and targeted pest control strategies.

## 1. Introduction

The fall armyworm, *Spodoptera frugiperda* (J.E. Smith) (Lepidoptera: Noctuidae), which results in substantial economic losses globally, features a broad host spectrum, robust migratory ability, and high reproductive capacity [1,2,3,4]. The species was first recorded as an injurious pest and named by Smith & Abbott in 1797 [5], and has had severe “marching-worms”—caused outbreaks that spread across almost the entire U.S. from 1856 onward [6,7]. Additionally, the first record of *S. frugiperda* in West and Central Africa dates back to early 2016 [8]. After that, confirmation was made that the species had spread and multiplied across all sub-Saharan countries [9], along with various Asian countries such as Pakistan [10], Nepal [11], South Korea, and Japan [12,13]. In China, this insect spread to Yunnan in 2019 [14], and it is projected that, in 2025, the area of corn in the middle and late growth stages afflicted by *S. frugiperda* across China will reach 13 billion m^2^ [15]. Recognized as an A1 Quarantine Pest in the European Union (EU) in 2020 [16], this pest was identified by the Ministry of Agriculture and Rural Affairs of the People’s Republic of China (MARA) as both a First-category Crop Disease and Pest and a Key Managed Invasive Species in 2022 and 2023 [17,18].

The successful global invasion of *S. frugiperda* can be largely attributed to its ability to overcome diverse environmental challenges, a capacity increasingly linked to its intimate association with gut microbiota. In general, microbial symbionts exhibit far greater diversity within the insect gut lumen than in host cells or on the cuticle [19]. In recent years, interest in insect gut microbiota has grown substantially, as these microbial communities are known to help maintain gut homeostasis and significantly influence host development, reproduction, nutrition, immunity, and other vital phenotypes [19,20,21,22]. In the case of *S. frugiperda*, shifts in the composition and diversity of larval gut microbiota could lead to microbial imbalance, which in turn impacts both energy metabolism and metabolic stability [23]. Such functional connections underscore the ecological and physiological relevance of gut microbes in this major pest.

Given the distinct and unique roles of gut microbiota in regulating the survival, reproduction, and adaptive capacity of *S. frugiperda*, this review will systematically focus on five core dimensions to elaborate on the interactions between the pest and its gut symbionts: First, regarding the composition and diversity of gut microbiota, it will clarify the dominant microbial taxa in different developmental stages of *S. frugiperda* and analyze how environmental factors (e.g., host plants and pesticide exposure) drive variations in microbial community structure. Second, for techniques for exploring gut microbiota, it will summarize both traditional methods and modern high-throughput technologies, while comparing their advantages in resolving microbial taxonomic details and functional potentials. Third, in terms of physiological functions modulated by gut microbiota, it will elaborate on specific regulatory roles. Fourth, regarding the potential values of gut microbiota in controlling *S. frugiperda*, it will discuss feasible application directions, including designing targeted strategies to disrupt gut microbial homeostasis. Finally, we also propose future prospects and outline challenges for research on the gut microbiota of *S. frugiperda*, aiming to further advance this research area.

A deeper and more comprehensive understanding of the taxonomic structure and functional roles of the gut microbiome in *S. frugiperda* will not only help decipher the underlying mechanisms of its rapid global invasion (such as how gut symbionts promote adaptation to new host plants or climatic zones) but also expect to reveal potential targets for novel microbiome-based management strategies. These targets may include key symbiotic microbes that are indispensable for the pest’s survival, or specific metabolic products of the gut microbiome that regulate pest behavior, which could provide new ideas to break through the limitations of traditional chemical pesticide control and achieve more sustainable pest management.

## 2. Composition of Gut Microbiota in *Spodoptera frugiperda*

Three major regions make up the insect gut: the anterior midgut, posterior midgut, and hindgut [24]. The gut microbial community of insects comprises a variety of microorganisms, including protists, fungi, archaea, viruses, and bacteria, with bacteria representing the dominant group [25,26]. In recent years, the number of studies on the gut microbial diversity of *S. frugiperda* has been on the rise [27,28,29]. Studies have shown that the gut microbiota of *S. frugiperda* is predominantly composed of the phyla Firmicutes and Proteobacteria [27,30], with smaller proportions of Bacteroidetes and Actinobacteria [30]. Firmicutes and Proteobacteria play essential roles in nutrient acquisition, maintenance of gut homeostasis, and modulation of host immune function [31,32,33]. A high abundance of these bacterial phyla has also been reported in other lepidopteran insects, such as *Choristoneura fumiferana* [34], *Plutella xylostella* [35], *Busseola fusca* [36], and *Ostrinia nubilalis* [37].

At the family level, Enterobacteriaceae and Enterococcaceae stand out as two key microbial groups in *S. frugiperda* [29]. This finding aligns with comparative analyses across 30 Lepidoptera species, which recognize both families as core components of the lepidopteran gut microbiota [38], with the function of assisting the host insects in digestion and detoxification processes. At the genus level, in all samples and across all developmental stages of *S. frugiperda* collected from corn fields in Dali City, Yunnan Province, China, *Enterococcus* (within Enterococcaceae) is the most abundant microbiota, accounting for 51.1%, followed by *Enterobacter* (within Enterobacteriaceae) at 23.2% [39]. Previous studies have revealed that *Enterococcus* is associated with the degradation of alkaloids and latex, as well as the detoxification of plant secondary metabolites [40,41]. Moreover, *Enterobacter* contributes to vitamin and pheromone synthesis, degradation of plant compounds, and nitrogen fixation [42]. Other bacterial genera (*Klebsiella*, *Providencia*, *Acinetobacter*, *Pseudomonas*, *Ralstonia*, *Sediminibacterium*, and *Serratia*) were verified in an earlier study of *S. frugiperda*; most of these may be beneficial for facilitating digestive processes and immunity of host insects within the gut [39,43]. Compared to gut microbiota in *S. frugiperda*, the bacterial diversity in *Diaphania pyloalis* was significantly simpler, with *Wolbachia* as the principal component [44]. For *Bombyx mori*, high-throughput sequencing has identified Proteobacteria, Firmicutes, Actinobacteria, and Bacteroidetes as the dominant taxa [44]. In contrast, *Enterococci* and *Clostridium* serve as the core gut bacteria in both *S. littoralis* larvae and *Helicoverpa armigera* larvae [45].

In parallel, the intestinal tract of *S. frugiperda* harbors a fungal community that develops a close-knit and sophisticated interaction with the host. The representative genera include *Candida*, *Sarocladium*, *Sporobolomyces*, *Moesziomyces*, and *Mucor* [46]. As vital symbionts in insect intestines, novel *Candida* species have been isolated from the guts of insects such as beetles [47]. *Sarocladium,* which has been isolated from the intestinal tracts of termites, exhibits cellulolytic and xylanolytic activities [48]. *Sporobolomyces* is widely distributed in natural environments, characterized by notable ecological adaptability [49]. *Moesziomyces* can break down certain types of plastics and serves as a primary producer of mannose erythritol lipid (MEL), an important surfactant belonging to the glycolipid class [50]. The genus *Mucor*, commonly found in diverse habitats ranging from soil and feces to air, secretes highly active proteases and contributes to the moderate degradation of soybean protein, among other functions [51].

Although some studies have isolated viruses (including baculoviruses and Reoviridae) from whole bodies of *S. frugiperda* that can be potentially used for pest control [52], the proportion of viruses in the gut of *S. frugiperda* is extremely low [26], making it extremely difficult to routinely isolate them for biological control.

## 3. Diversity of *Spodoptera frugiperda* Gut Symbionts

In herbivorous insects, the composition of gut microbial communities is influenced by a range of biotic and abiotic factors [53,54,55]. Evidence has shown that the diversity of gut microbiota in *S. frugiperda* can be modulated by multiple variables, including different diets (host plants and artificial diet) [25,26,56,57,58], host developmental stage [27], environmental stressors (e.g., dryness and humidity) [43], and sex [39] (Table 1). Understanding how these factors shape the gut microbiome of *S. frugiperda* can provide critical insights into the ecological adaptability of this global pest.

Diet stands as the primary factor capable of rapidly and significantly altering the relationship between insect hosts and their gut microbiota, and it profoundly impacts the composition, taxonomy, and function of microbial communities—an observation that underscores the remarkable plasticity of gut microbiota [62,63]. Compared with *S. frugiperda* reared on a field-collected diet, those fed an artificial diet exhibited higher microbial diversity. In contrast, the relative abundance of Firmicutes was higher in the field diet group than in the lab diet group [58]. This discrepancy might lie in the high cellulose and hemicellulose in leaf samples—these components increase the host’s reliance on Firmicutes for efficient polysaccharide digestion [64].

Owing to its high polyphagy and an extensive host range, *S. frugiperda* larvae can consume more than 300 plant species, including gramineous crops such as maize, wheat, rice, sorghum, as well as vegetable crops such as oilseed rape, pepper, potato, and other crops of economic significance [1,46,65]. The microbial abundance and diversity linked to various plants such as maize, rice, cotton, wheat, potato, tomato, soybean, pepper, millet, oilseed rape, pakchoi, purple cabbage, and wild oat were different, indicating that they supply different nutrients to support *S. frugiperda* development [57,59,66,67,68]. Furthermore, the host plant has been found to alter not just the bacterial community but also the fungal community of *S. frugiperda* [69]. Similarly, in other economically significant pests (including *S. littoralis*, *Agrotis ipsilon*, *Busseola fusca*, *Helicoverpa armigera*, *Heliothis virescens*, *Manduca sexta*, *Ostrinia nubilalis*, and *Plutella xylostella*), the makeup of microbiota also varies between polyphagous and oligophagous species [70].

The gut microbial community in *S. frugiperda* undergoes dynamic changes throughout its complete metamorphic life cycle. During larval development, microbial richness is highest in early instars but declines significantly from the fourth instar onward, remaining low thereafter. These shifts result in three distinct clustering patterns corresponding to early, middle, and late developmental stages. It is hypothesized that the gut bacterial community may undergo a “screening” process during larval growth, which selectively retains microorganisms that are better adapted to and support the host’s physiological requirements [71]. Exposure to insecticides, as well as dry and rainy seasons, significantly reshaped the gut microbiome of fall armyworm, causing a chain reaction of changes in community diversity, assembly patterns, as well as bacterial abundance [43,72]. However, the intervention of antibiotics disrupts the host–microbiome balance, leading to a process of disruption and incomplete recovery, thereby altering the insect’s insecticide resistance phenotype [73].

In *S. frugiperda*, sex-specific differences exist in gut microbial communities, which are reflected not only in composition but also in functional contributions to host physiology [29]. Unlike the relatively stable microbial profiles in male individuals, female-specific microbial assemblages show unique structural characteristics, which may be closely tied to their distinct hormonal fluctuations and physiological demands across life stages [39]. Metabolically, females exhibit consistently higher activity levels than males, a trait associated with their elevated nutritional and energy demands, which support energy-intensive processes such as long-distance migration, ovarian development, and reproduction. To meet these elevated nutritional and energetic demands, female *S. frugiperda* may rely on a more abundant and functionally diversified gut bacterial community, enhancing nutrient acquisition efficiency. This microbial enrichment not only fulfills the female’s own metabolic requirements but also supports the transfer of essential nutrients to offspring. Thus, the female-specific gut microbiome constitutes an adaptive trait that balances maternal physiology with reproductive investment, contributing to the species’ population persistence [39].

## 4. Methods for Exploring Gut Microbiota

**Figure 1:**
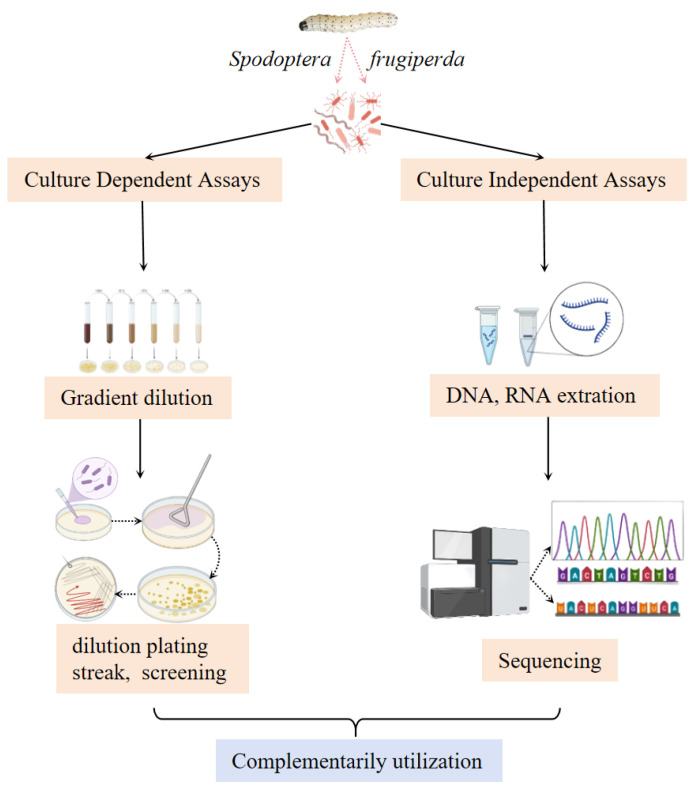
Methods for exploring gut microbiota of *Spodoptera frugiperda*.

Characterizing gut microbiota and elucidating their functional roles are essential for understanding microbial–host interactions in insects. The approaches for taxonomic identification of gut microorganisms fall into two main categories: culture-dependent and culture-independent methods (Figure 1), with the choice often influenced by sample origin and research objectives [74,75]. While culture-dependent methods are effective for isolating abundant or readily culturable species, they often overlook less dominant or fastidious microbes. In contrast, culture-independent techniques, such as high-throughput sequencing, provide broader coverage and a more comprehensive perspective of microbial diversity [76]. For studies related to pest outbreak monitoring or risk assessment, an integrated approach combining both strategies is recommended to maximize taxonomic resolution and functional insight.

### 4.1. Culture-Dependent Methods

Culture-dependent methods focus on the enrichment of known microbial groups through customized media and controlled incubation [77], a strategy that promotes the growth of target organisms while suppressing non-target species. The resulting isolates can be further analyzed via phenotypic characterization, genetic sequencing, or functional assays to deeply elucidate their biological properties [78]. This method’s quantitative comparisons of microbial abundance may overestimate dominant culturable taxa and underestimate unculturable ones, so rigorous interpretation is required when linking isolate data to the actual gut microbiome structure. Although this approach has inherent limitations in capturing unculturable microbes that may account for a large proportion of the gut microbiome, it remains indispensable in early-stage research, particularly for studies focused on functional microbial strains with direct application value in pest management.

Traditional microbial isolation and purification methods—encompassing dilution plating, streak culture, and selective medium screening—have been extensively employed to study the gut microbiota of *S. frugiperda*. Population samples were collected from major infestation regions across southern China, such as Yunnan, Chongqing, and Hunan provinces, where the insect exhibits high population density and strong agroecological adaptability [46,79,80]. Using culture-dependent methods, diverse bacterial (e.g., *Klebsiella*, *Enterococcus*) and fungal (e.g., *Candida*, *Sporobolomyces*) taxa have been successfully isolated from the *S. frugiperda* gut. These isolated microbes not only enrich the inventory of known *S. frugiperda* gut symbionts but also lay a material foundation for subsequent functional verifications. However, the diversity of isolated microbes may not reflect the true community composition in the insect gut, limiting the comprehensiveness of functional prediction.

Notably, beyond microbial isolation, the traditional culture-dependent method also provides a feasible and cost-effective tool for the preliminary investigation of the mechanisms behind *S. frugiperda*’s response to biotic and abiotic stresses. For instance, by culturing gut microbes under simulated chemical stress (e.g., exposure to insecticides), researchers can initially screen for stress-tolerant microbial strains and infer their potential mechanisms in enhancing the insect’s stress resistance [72,81]. While this screening strategy is efficient for identifying candidate functional strains, its limitation lies in the disconnect between laboratory stress simulation and field conditions—strains performing well in vitro may not exert the same effect in vivo, within the complex gut environment. To address this, data from culture-dependent stress assays should be validated by complementary in vivo experiments (e.g., microbe reintroduction into axenic insects) to confirm functional relevance.

### 4.2. Culture-Independent Methods

Culture-independent methods are highly recommended for the initial taxonomic profiling of insect gut microbial communities [82], as a substantial proportion of these microorganisms in such communities cannot be cultivated in vitro due to fastidious nutritional requirements, sensitivity to laboratory environmental conditions, or dependence on host interactions.

In previous studies, 16S rRNA metagenomics has been widely employed to characterize the gut microbial community structure and diversity of *S. frugiperda*. This high-throughput approach has revealed stage-specific microbial assemblages across different developmental stages (from egg to larva, pupa, and adult), suggesting tailored functional roles in supporting physiological needs during development [27,29,39]. However, taxonomic profiling relies heavily on reference databases—microbes without matched sequences may be misclassified or labeled as “unidentified”, leading to underestimated true diversity. Furthermore, culture-independent analyses have been applied to assess how factors such as diets, environmental stressors, host plant identity, and agrochemicals influence the composition and diversity of gut microbiota in this species [28,40,43,56,57,58,59,60,61,83]. A key drawback is the lack of standardization in experimental design, making cross-study comparisons challenging and reducing the reproducibility of findings.

Unlike 16S rRNA gene sequencing, which targets taxonomic composition, metatranscriptomics provides deep functional insights by capturing genome-wide transcriptional activity within microbial communities [52]. This approach enables profiling of actively expressed genes involved in key metabolic processes such as carbohydrate fermentation, amino acid biosynthesis, and lipid metabolism, while also identifying which microbial taxa are functionally relevant under specific physiological or environmental conditions [84,85].

## 5. Function Modulated by Gut Microbiota

Symbiotic gut microbiota significantly enhance the environmental adaptability and geographical expansion of *S. frugiperda* by sustaining intestinal homeostasis and influencing key host physiological traits [22] (Table 2). Specifically, these microbes facilitate the breakdown of complex nutrients and plant secondary metabolites, thereby promoting development [23]. They also modulate host immune responses, improving resistance to pathogens and environmental stressors in new habitats [86,87]. Through these functions, beneficial gut microbes play an integral role in supporting the pest’s successful invasion and establishment across diverse ecosystems; nevertheless, studies on the related mechanisms are relatively rare.

### 5.1. Role of Gut Symbionts in Xenobiotic Degradation In Vitro

In modern agricultural systems, chemical pesticides remain the primary strategy for pest control, playing a crucial role in safeguarding crop yield and quality. However, the extensive and prolonged application of these chemical compounds has driven the evolution of adaptive resistance traits in economically significant pests such as *S. frugiperda*. This pest species has developed notable resistance to a range of commonly used insecticides, including lufenuron, chlorantraniliprole, indoxacarb, lambda-cyhalothrin, and *Bacillus thuringiensis* endotoxins, significantly reducing the effectiveness of chemical control measures and posing serious challenges to sustainable crop protection [60,73,87].

Recent research has shed light on the critical contribution of gut microbes in *S. frugiperda* to its chemical resistance, operating through both direct metabolic degradation and indirect physiological modulation. For instance, isolated gut symbiotic strains have demonstrated the ability to dechlorinate and degrade polyvinyl chloride (PVC). Specifically, *E. casseliflavus* EMBL3 encodes NAD-dependent oxidoreductase that dechlorinates PVC polymer [88]. Additionally, *Klebsiella* C3 alters the host’s metabolic profile, affecting key detoxification pathways and thereby modulating *S. frugiperda*’s sensitivity to lufenuron, highlighting the diverse detoxification capabilities of the gut microbiome [87]. These findings broaden our understanding of resistance evolution beyond host-centric mechanisms and reveal novel potential targets for pest management [90,91,92,93].

### 5.2. Role of Gut Symbionts in Altering Host Tolerance In Vivo

The gut microbiota regulates the physiological functions of insect hosts. Accumulating evidence has established clear links between the structure and function of the microbiota and key insect life-history traits, particularly development and reproduction. Elevated reproductive capacity is critical for invasive insects like *S. frugiperda* to successfully colonize new habitats, enabling rapid population expansion and outbreaks that often cause severe impacts on agricultural ecosystems [94,95]. Throughout the insect’s molting and metamorphosis processes, its gut microbial communities undergo considerable restructuring. However, core bacterial genera such as *Enterococcus* persist consistently, underscoring their indispensable role in sustaining *S. frugiperda*’s development and fitness [29,39].

Functional verification studies have confirmed the regulatory role of gut symbionts. Gut bacteria in *S. frugiperda*, such as *Enterococcus* and *Enterobacter*, have been shown to facilitate host growth and development even under nutrient-deficient dietary conditions [96]. Furthermore, microbiota-depleted *S. frugiperda* exhibited impaired ovarian development and fecundity, while reinoculation with specific bacterial strains restored reproductive output to normal levels [78]. These findings, consistent with studies on other insect species such as *Zeugodacus tau* and *Henosepilachna vigintioctopunctata* [97,98], collectively confirm that gut microbiota is indispensable regulators of *S. frugiperda*’s reproductive performance and developmental fitness, and providing a theoretical basis for exploring microbiota-based pest management strategies.

### 5.3. Interactive Relationships Involving Gut Symbionts

Beyond facilitating detoxification and development, microbial symbionts of *S. frugiperda* also modulate induced defense responses in host plants [99]. Specifically, insect-associated symbiotic bacteria can act as ecological decoys, triggering plant immune responses typically directed against microbial pathogens rather than herbivores. This misdirection effectively suppresses the activation of plant defense mechanisms, thereby creating a more favorable feeding environment for *S. frugiperda* larvae and enhancing their development [100]. Therefore, targeted manipulation of the herbivore’s microbiota presents a potential strategy to disrupt these interactions, ultimately impairing the insect’s performance on defensive host plants [101,102].

The interactive relationships mediated by the gut microbiome of *S. frugiperda* extend across multiple trophic levels, resulting in complex ecological effects. While substantial research has characterized lepidopteran oral secretions regulating plant defense induction, the involvement of microbes in these interactions has been largely overlooked [103]. On one hand, gut bacteria of *S. frugiperda*, such as the JA defense-suppressing strains *Pantoea ananatis* and Enterobacteriaceae-1 (genus *Serratia/Rahnella*), can be secreted via the insect’s regurgitant or frass to directly modulate defense responses in tomato plants, thereby improving the insect’s performance [86]. On the other hand, certain plant defenses can compromise gut barrier integrity; this allows gut microorganisms to translocate into the body cavity, thereby amplifying the detrimental effects of defense compounds on the insect host [89]. Furthermore, parasitism can induce cascading effects on microbial communities across trophic levels, indicating that gut bacteria are integral components of the complex interactions among parasitoids, herbivores, and their host plants [100].

## 6. Techniques for Insect Pest Control

**Figure 2:**
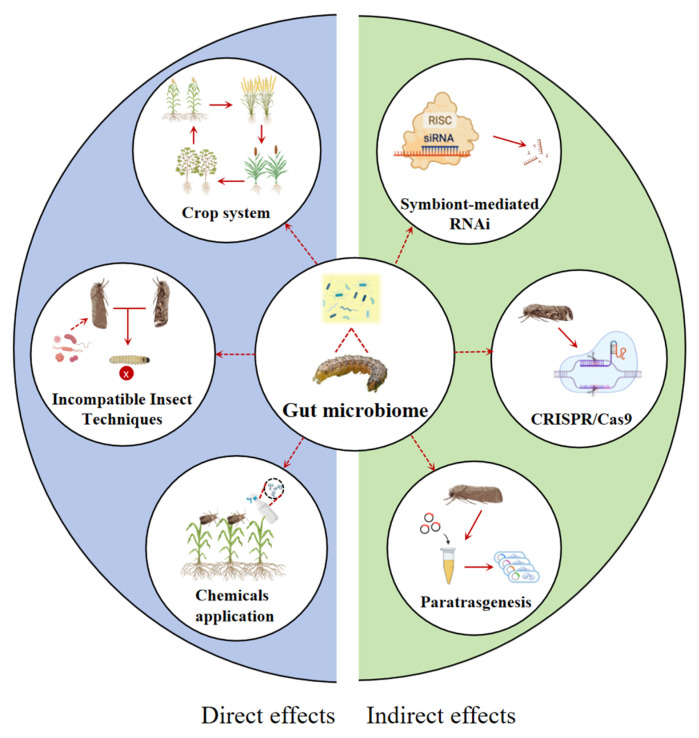
The guide of gut microbiota for *Spodoptera frugiperda* pest management.

The symbiotic microbiome provides insects with holistic benefits, influencing development, nutrition, reproduction, defense against pathogens, and detoxification processes [19,104,105,106,107,108]. Given the recognized potential of insect gut endosymbionts in biotechnology and sustainable pest control, research on insect gut microbes holds significant promise for innovative management strategies [109]. In the case of *S. frugiperda*, a globally invasive pest of maize and other crops, targeted manipulation of its gut microbiota, when integrated with existing control strategies, may enhance suppression efficacy [110,111,112]. Modern integrated pest management (IPM) entails the appropriate integration of diverse pest control strategies [113]. The gut microbiota represents a promising regulatory target and could be leveraged to control *S. frugiperda* through the combined application of incompatible insect techniques (IIT), genetic modification, and application of chemicals [114,115,116] (Figure 2).

### 6.1. Incompatible Insect Techniques (IIT)

Targeting the gut microbiota of *S. frugiperda* presents a sustainable alternative to conventional chemical pesticides, which are major contributors to ecological contamination and rapid pest resistance. The Incompatible Insect Technique (IIT) leverages endosymbionts to manipulate insect populations by inducing phenotypic effects such as cytoplasmic incompatibility, parthenogenesis, and feminization [117]. Specifically, cytoplasmic incompatibility (CI) relies on symbiont infection in males (but not females), triggering abnormal mitosis during the first embryonic division and resulting in embryonic mortality; parthenogenesis induction involves symbionts initiating the development of unfertilized eggs, producing female-only offspring with reduced genetic diversity; feminization occurs when symbionts interfere with the male sex-determination pathway, leading to genetic males developing as functional females and thereby skewing population sex ratios. The core principle has been successfully demonstrated in several insect models, including *Drosophila melanogaster* (with *Acetobacter cerevisiae*), the Mediterranean fruit fly *Ceratitis capitata* (with *Wolbachia*-induced CI), and the mosquito *Culex quinquefasciatus* (with CI) [116,117,118,119,120,121]. These established precedents validate the core principles of IIT and provide a framework for adapting this approach to the biological specificities of *S. frugiperda*.

While promising, the application of IIT to *S. frugiperda* is not straightforward. A significant limitation of many foundational studies is their focus on laboratory or confined field conditions, which may not accurately predict efficacy in the complex, open-field environments [122]. The successful establishment of infection in a naive population requires high-fidelity maternal transmission and a significant fitness cost to uninfected individuals, conditions that are difficult to guarantee [123]. Furthermore, the technique’s success is highly dependent on the specific host–symbiont combination; a strain that induces strong CI in one species may be ineffective or not establish in another [124]. A critical failure point in any IIT program would be the incomplete maternal transmission of the symbiont, leading to the rapid emergence of uninfected, fertile individuals that could collapse the suppression effect.

Proposed Experimental Pipeline for IIT in *S. frugiperda*:

#### 6.1.1. Symbiont Discovery and Screening

Systematically characterize the native microbiome of *S. frugiperda* from different geographical populations to identify candidate endosymbionts.

#### 6.1.2. Laboratory Validation 

Microinject candidate symbionts into aposymbiotic (symbiont-free) *S. frugiperda* eggs. Key monitoring metrics include maternal transmission rate, CI penetrance, and host fitness costs.

#### 6.1.3. Contained Field Trial (e.g., Large Field Cages)

Release infected males into cages containing a known density of uninfected wild-type insects. The primary success metric is the suppression of the F1 generation population compared to control cages.

### 6.2. Genetic Modification of Symbionts

The gut microbiota is a reservoir of genes essential for host fitness, making it a target for precision genetic manipulation. Strategies include CRISPR/Cas9-mediated gene knockout, paratransgenesis, and symbiont-mediated RNAi (SMR) [125]. The precision of these strategies enables rigorous validation of gene functions while minimizing off-target effects, thereby establishing a foundation for developing precise and environmentally safe pest control tools.

The CRISPR/Cas9 and paratransgenesis systems allow for the functional disruption of microbial genes critical for nutrient provisioning or detoxification, thereby inducing dietary deficiencies or increasing pesticide susceptibility in the host. They open innovative avenues for pest control through the functional manipulation of symbiotic microbes [126,127,128,129,130]. For instance, in the mosquito *Aedes aegypti*, the Scarless Cas9-Assisted Recombineering (no-SCAR) system was used to knock out the *ompA* gene in the gut symbiont *Cedecea neteri*, which impaired biofilm formation [131]. However, challenges like off-target effects, inefficient delivery to non-model symbionts, and the fitness cost imposed on the engineered microbe remain significant hurdles [126].

Additionally, Symbiont-Mediated RNAi (SMR) employs engineered bacteria to deliver host-specific double-stranded RNA (dsRNA), a technique validated in multiple insect pests [132,133]. This method overcomes key limitations of conventional RNAi, such as environmental instability of dsRNA and poor oral uptake, enabling sustained and efficient gene silencing within the host. Nevertheless, the presence of gut nucleases and the efficiency of the insect’s RNAi machinery are the primary barriers to effective RNAi [132].

The release of engineered symbionts poses serious and plausible ecological risks. The primary concern is horizontal gene transfer (HGT) of the transgene to non-target microorganisms in the gut of other insects or in the soil, potentially altering ecological functions or conferring unknown advantages [134]. Pre-release risk assessment must therefore include rigorous host-specificity testing and investigations of HGT potential in complex microbial communities. Furthermore, the complexity of insect microbiomes and their context-dependent interactions with the host presents a significant challenge in predicting the ecological outcomes of releasing engineered symbionts [135].

The deployment of engineered symbionts is tightly constrained by an evolving and often stringent regulatory landscape. Internationally, the Cartagena Protocol on Biosafety [136] governs the transboundary movement of Living Modified Organisms (LMOs). Nationally, countries such as China (Measures for the Safety Administration of Genetic Engineering) and the European Union (with its stringent GMO directives) operate hierarchical approval systems that require extensive Environmental Risk Assessment (ERA) [137,138]. Beyond regulation, public acceptance is a critical barrier. The concept of releasing “genetically modified microbes” faces significant societal skepticism, often amplified by a lack of public understanding and low trust in regulatory institutions. Successful deployment will require transparent science communication and proactive stakeholder engagement.

Proposed Experimental Pipeline for Engineered Symbionts:

#### 6.2.1. Engineer Symbiont 

Use engineered strategies to knockout a gene for an essential nutrient in a culturable gut symbiont of *S. frugiperda*.

#### 6.2.2. Assess Efficacy 

Re-introduce the knockout strain into axenic *S. frugiperda* and monitor for the predicted fitness deficit.

#### 6.2.3. Risk Assessment

Host Range Testing: Feed the engineered symbiont to non-target Lepidopterans and other insects to assess colonization ability. HGT Potential: Co-culture the engineered symbiont with a diverse microbial community in vitro and use selectable markers to screen for gene transfer events. Persistence Monitoring: In contained trials, track the environmental persistence of the engineered strain outside the host.

### 6.3. Chemicals Applied in Field Management

In agricultural field production, strategically designed crop rotation and intercropping systems, when properly implemented, can effectively mitigate pest issues in an environmentally sustainable manner [139]. Beyond such agronomic practices, chemical-based intervention strategies have also been explored. Notably, a review evaluated experimental and theoretical studies on mixtures (herbicides, insecticides, fungicides, and antibiotics), where each component was applied at its full label rate, have confirmed that such mixtures are an optimal strategy for resistance management [140,141]. A chemical in a mixture with a different mode of action can slow resistance evolution [142]. Additionally, bioactive molecules such as antimicrobial peptides (AMPs) have emerged as alternative tools for targeted insect symbiont control. The mechanism relies on electrostatic interactions: the positively charged AMPs bind to the negatively charged microbial cell membrane, disrupting the structural integrity of the cell wall and impairing microbial viability [143]. This mode of action has been validated in multiple insects such as *Anopheles gambiae* and *A. aegypti* [144,145], supporting the potential of AMPs as a precision tool for microbiome-based pest management.

While theoretically sound for targeting a single pest, this approach is highly inefficient in polyphagous pests like *S. frugiperda* that infest multiple crop growth stages, as components may be redundant or target different, non-co-occurring species. This leads to a disproportionate increase in environmental load, cost, and regulatory complexity without a proportional gain in efficacy [146,147]. Crucially, mixtures can accelerate the evolution of cross-resistance if a single metabolic detoxification mechanism confers tolerance to both chemicals. Therefore, rotating modes of action remains a more practical and lower-risk resistance management strategy [148]. Additionally, the use of antibiotics for field-based symbiont control is non-targeted, devastating the beneficial microbiomes of soil, water, and non-target organisms. This makes their field application for pest control fundamentally infeasible.

Although bioactive molecules offer a more targeted approach, their specificity for pest symbionts over beneficial microbes needs rigorous testing. Beyond chemicals, agroecological practices such as push–pull intercropping and strategic crop rotation are effective in managing *S. frugiperda* by directly altering the habitat and reducing host plant availability. These strategies represent the most sustainable and low-risk foundation for an integrated management program.

## 7. Future Perspectives and Challenges

**Figure 3:**
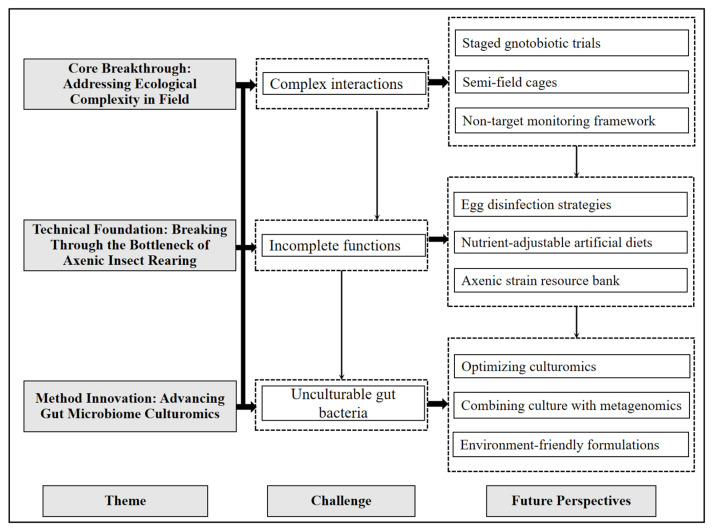
Roadmaps that bridge key lab findings to realistic field tests.

Research on the interactions between *S. frugiperda* and its gut microbes provides valuable insights into insect-symbiont coevolution, thereby enriching the theoretical foundations of microbial ecology and insect physiology. However, despite the promising potential of utilizing *S. frugiperda*’s gut bacteria for pest management, significant challenges remain in their practical application (Figure 3):

### 7.1. Core Breakthrough: Addressing Ecological Complexity in Field

A critical challenge lies in translating laboratory-based findings on the gut microbiome to field-scale pest control. In agricultural ecosystems, insect-gut microbe symbioses are inherently influenced by complex networks of abiotic (e.g., temperature and precipitation) and biotic (e.g., non-target insects and plant microbiomes) factors. This complexity often leads to discrepancies between conclusions drawn from controlled laboratory studies and results observed in practical applications [149]. To bridge this gap, practical measures include staged gnotobiotic trials (verifying microbial functions in axenic environments first, then introducing ecological factors), semi-field cages (replicating farmland conditions to test microbial control effects), and non-target monitoring frameworks (using high-throughput sequencing to track non-target organism dynamics and evaluating ecosystem impacts via long-term plots). Implementing such measures is crucial for developing microbial control strategies that are both ecologically sustainable and effective in practical agricultural settings.

### 7.2. Technical Foundation: Breaking Through the Bottleneck of Axenic Insect Rearing

A major obstacle in developing microbiome-targeted pest control is the incomplete understanding of gut bacterial functions. Axenic rearing systems are designed to address this by enabling the deconstruction and reassembly of microbial communities [150,151]. However, establishing axenic insect lines is technically challenging, limiting functional study scalability. We recommend the following practical solutions: refining egg disinfection strategies (e.g., sequential treatment with sodium hypochlorite and ethanol) to maintain hatching rates, developing nutrient-adjustable artificial diets, and establishing a centralized repository for axenic strains, accompanied by standardized records of key biological parameters. Collectively, these measures would strengthen the technical foundation for elucidating the functional roles of gut microbes.

### 7.3. Method Innovation: Advancing Gut Microbiome Culturomics

Elucidating the functional roles of individual gut microbial taxa is hindered because most gut bacteria are unculturable under traditional laboratory conditions, where standard media and static environments fail to replicate the in vivo gut microclimate [152,153]. While high-throughput culturomics-which integrates diverse media and environmental simulation-has enabled the cultivation of previously inaccessible strains, its large-scale application remains constrained by technical and logistical challenges. Practical measures to address these limitations include: optimizing culturomics through the development of gut-mimicking media with physiologically relevant pH and oxygen gradients; integrating culturomics with metagenomics by using sequencing data to predict functional genes and guide targeted isolation; and formulating microbial formulations within protective microcapsules to enhance their persistence in field applications.

The continued advancement of high-throughput sequencing and multi-omics technologies (e.g., metagenomics, metatranscriptomics), will enable systematic profiling of the gut microbial community in *S. frugiperda*, including its composition, structure, and function. Integrating multi-omics data to construct host–microbe interaction networks, complemented by laboratory and field validation, will be central to translating gut microbiome research into practical *S. frugiperda* control strategies. Enhanced international collaboration and resource sharing will be crucial to accelerate this process, facilitating the translation of research findings into effective management tools for *S. frugiperda—*a prominent transboundary pest.

## 8. Conclusions

This review synthesizes current knowledge of the gut microbiota in *S. frugiperda*, emphasizing its pivotal role in host physiology and identifying host–microbe interactions as promising targets for novel pest management strategies. The gut microbiome represents a unifying target for integrating multiple control strategies, especially eco-friendly approaches, to compromise the pest’s fitness. The validation of insect symbionts as critical targets firmly establishes the gut microbiota of *S. frugiperda* as a foundation for developing sustainable and effective population suppression tools.

Specific microbial taxa merit in-depth investigation to accelerate practical applications. Among *Firmicutes*, *Enterococcus* spp., which are consistently identified as dominant members of the gut microbiota, should be the focus of functional characterization, elucidating the metabolic pathways involved in xenobiotic degradation or beneficial metabolite production by these strains could inform the design of synergistic pest control strategies. Within Proteobacteria, insecticide-degrading taxa such as *Pseudomonas* and *Sphingomonas* spp. warrant detailed genomic and enzymatic studies to identify key functional genes and develop engineered microbial strains with enhanced degradation capabilities for field deployment. Additionally, potentially important taxa, including cellulolytic and nitrogen-fixing bacteria (e.g., *Cellulomonas* and *Azospirillum* spp.), should be systematically screened for their ability to modulate host feeding behavior and nutrient utilization, as disrupting these interactions could provide novel avenues for pest control. Finally, rigorous assessments of the ecological risks, off-target effects, and regulatory compliance of microbiome-based interventions are essential to ensure their safe and sustainable integration into agricultural systems, thereby contributing to environmentally friendly and cost-effective management of *S. frugiperda* worldwide.

## Figures and Tables

**Table 1:** Diversity of gut microbiota in *Spodoptera frugiperda.*

Variates	Dominant Gut Microbiota	References
Sugarcane, maize, onion	Acidobacteriia, Deltaproteobacteria, Clostridia, Alphaproteobacteria, Bacteroidia, Actinobacteria, Bacilli, etc.	[25]
Maize, hairy vetch	*Enterococcus*, *Bacillus*, *Klebsiella*, *Acinetobacter*, *Pseudomonas*, etc.	[56]
Corn, wild oat, oilseed rape, pepper, an artificial diet	Enterococcaceae, Muribaculaceae, Enterobacteriaceae, Lachnospiraceae, etc.	[59]
Developmental stage	*Enterococcus*, *Klebsiella*, *Acinetobacter*, *Pseudomonas*, *Lactobacillus*, *Streptococcus*, etc.	[29]
Female and male	*Enterococcus*, *Enterobacter*, *Providencia*, *Ralstonia*, *Acinetobacter*, etc.	[39]
Laboratory population	Actinobacterica, Bacteria, Bacteroidetes, Firmicutes, Proteobacteria, Thaumarchaeota, etc.	[40]
Field population	Actinobacterica, Bacteria, Bacteroidetes, Firmicutes, Proteobacteria, etc.	
Dry and rainy season	*Enterobacter*, *Enterococcus*, *Klebsiella*, *Microbacterium*, *Ralstonia*, *Turicibacter*, etc.	[43]
*Bacillus thuringiensis* exposure	*Enterococcus*, *Weissella*, *Ileibacterium*, *Ralstonia*, *Dubosiella*, etc.	[60]
Exposure to broflanilide, spinosad and indoxacarb	*Acinetobacter*, *Pelomonas*, *Rhodococcus*, *Ralstonia*, *Bacteroides*, etc.	[61]

**Table 2:** The category and function of some important symbiotic microbiomes of *Spodoptera frugiperda*.

Symbiont	Roles	References
*Enterococcus quebecensis* *Klebsiella michiganensis* *Enterobacter hormaechei*	Enhance reproduction	[78]
*Enterococcus* and *Weissella*	Influence metabolic homeostasis (energy production, metabolism, and the autophagy—lysosome) signal pathway	[23]
*Pantoea ananatis* Enterobacteriaceae-1	Modulate plant defense responses	[86]
*Klebsiella* C3	Promote host resistance against the toxic effects of lufenuron	[87]
*Enterococcus casseli* EMBL-3	Dechlorinate and degrade polyvinyl chloride	[88]
*Pseudomonas Stutzeri*	Lambda-cyhalothrin degradation	[72]
*Arthrobacter nicotinovorans*	Deltamethrin degradation	
*Leclercia adecarboxylata*	Chlorpyrifos ethyl degradation	
*Microbacterium arborescens*	Lufenuron degradation	
*Pseudomonas psychrotolerans*	Spinosyn degradation	
*Enterococcus*, *Klebsiella*, *Enterobacter*	Exacerbate the adverse effects of plant defenses on the insect	[89]

## Data Availability

No new data were created or analyzed in this study. Data sharing is not applicable to this article.

## Abbreviations

The following abbreviations are used in this manuscript:

AMPs	Antimicrobial peptides
CI	Cytoplasmic Incompatibility
dsRNA	Double-stranded RNA
ERA	Environmental Risk Assessment
EU	European Union
GMO	Genetically Modified Organism
HGT	Horizontal Gene Transfer
IIT	Incompatible insect techniques
IPM	Integrated pest management
LMOs	Living Modified Organisms
MARA	The Ministry of Agriculture and Rural Affairs of the People’s Republic of China
MEL	Mannose erythritol lipid
PVC	Polyvinyl chloride
